# Incidence and influencing factors of postoperative frailty in elderly prostate cancer patients: a cross-sectional study

**DOI:** 10.3389/fmed.2025.1649127

**Published:** 2025-10-14

**Authors:** Li Sun, Guoqin Ren, Qin Wang, Xumiao Zhang, Hongxia Hua, Yanglin Gu

**Affiliations:** ^1^Jiangnan University, Wuxi No. 2 People's Hospital, Wuxi, China; ^2^Department of Public Health, Wuxi No. 2 People's Hospital, Wuxi, China; ^3^Department of Oncology, Wuxi No. 2 People's Hospital, Wuxi, China; ^4^Department of Orthopedics, Wuxi No. 2 People's Hospital, Wuxi, China

**Keywords:** prostate cancer, radical prostatectomy, surgery, postoperative frailty, influencing factors

## Abstract

**Background:**

Postoperative frailty in prostate cancer patients represents a significant public health concern, as its persistent nature not only impedes recovery but also elevates the risk of adverse clinical outcomes. This study systematically evaluates the prevalence of postoperative frailty and investigates its determinants to establish an evidence-based foundation for developing targeted nursing interventions.

**Methods:**

From October 2024 to March 2025, a convenience sampling method was employed to select patients undergoing radical prostatectomy in the urology department of a certain tertiary hospital in Wuxi as the research subjects. General information questionnaires’ the VES-13 frailty scale, Karnofsky Performance Status Scale, Sleep Dysfunction Rating Scale, Social Support Rating Scale and Self-Rating Anxiety Scale were used for investigation. Univariate analysis and binary logistic regression were employed to analyze the influencing factors of frailty in postoperative prostate cancer patients.

**Results:**

Among 306 prostate cancer postoperative patients, 39.5% experienced frailty. Binary logistic regression showed that age, intraoperative blood loss, functional status, exercise frequency (≥3 times/week), anxiety, and social support level were the main influencing factors of frailty (*p* < 0.05).

**Conclusion:**

The incidence of postoperative frailty in prostate cancer patients is high and influenced by various factors. It is recommended that nursing staff actively assess and identify patients, taking proactive measures taking proactive measures to intervene and improve or reverse frailty status.

## 1 Introduction

Prostate cancer is a malignant tumor originating from the epithelial cells of the prostate. It ranks first in incidence among male urogenital system tumors (1). According to statistics from the International Agency for Research on Cancer (IARC), approximately 1.47 million new cases of prostate cancer are diagnosed annually, with an age-standardized incidence rate showing a significant upward trend (2). This poses a substantial challenge to global public health systems. Radical prostatectomy, as the primary treatment for localized prostate cancer, has experienced a continuous increase in clinical application rates due to advancements in surgical techniques (3). Frailty is a clinical syndrome characterized by a decline in physiological reserve and weakened stress response capacity associated with aging. It leads to dysfunction across multiple systems and increases bodily vulnerability (4). Clinical evidence indicates that the advanced age of prostate cancer patients, preoperative bladder outlet obstruction caused by tumor compression, long-term urinary difficulties, the direct trauma of surgery, and exacerbation of systemic inflammatory responses due to postoperative catheterization and pain collectively limit patient mobility, thereby increasing the risk of postoperative frailty (5). Postoperative frailty occurs under surgical stress, weakening the body’s recovery capacity and increasing the risk of complications while reducing tolerance to treatment. This not only impairs physical recovery and quality of life but also indirectly increases the demand for medical resources and imposes economic burdens on society (6). Therefore, investigating the current status of postoperative frailty and its influencing factors is of critical importance. Currently, research on postoperative frailty predominantly focuses on patients with digestive system tumors. In contrast, studies on prostate cancer patients primarily emphasize preoperative frailty assessment and prehabilitation strategies, with limited attention to postoperative frailty and its influencing factors. In light of this gap, the present study aims to examine the prevalence and determinants of postoperative frailty in prostate cancer patients, providing a foundation for identifying key modifiable factors, developing targeted intervention measures, and effectively reversing frailty.

## 2 Materials and methods

### 2.1 Study subjects

From November 2024 to March 2025, convenience sampling was used to select cancer patients who underwent radical prostatectomy at the urology department of a tertiary hospital in Wuxi City as the study population.

Inclusion criteria: Patients met the diagnostic criteria outlined in the 2023 edition of the “Chinese Quality Control Indicators for the Standardized Diagnosis and Treatment of Prostate Cancer” (7), and they are the first cases of diagnosis, and were clinically diagnosed with stage I-II; Had the first radical prostatectomy surgery; and were aged ≥60 years.

Exclusion criteria: Patients with severe cardiovascular, hepatic, renal, or cerebral diseases, or those with other malignant tumors; patients with severe mental disorders unable to communicate normally; patients with severe visual or auditory impairments; and patients who were frail prior to surgery.

The sample size was calculated based on Kendall’s principle (8), with the sample size being at least 5 ~ 10 times the number of independent variables. This study included 26 influencing factors. Considering a 20% error rate and missing data, the sample size was calculated to be between 156 and 312 cases. A total of 315 questionnaires were distributed in this study, with nine invalid questionnaires excluded, resulting in 306 valid questionnaires returned, yielding an effective return rate of 97.14%. This study has been approved by the hospital ethics committee (approval number: 2024-Y-306), and all participants provided informed consent and voluntarily participated in the study.

### 2.2 Research tools

#### 2.2.1 General information questionnaire

Designed by the researcher based on a review of domestic and international literature, combined with the results of a preliminary survey. Includes: age, BMI, marital status, educational attainment (highest level of education), smoking, alcohol consumption, frequency of daily exercise, method of medical insurance payment, past medical history (including chronic conditions), history of falls (within the past year), surgical history (within the past year), intraoperative blood loss, use of pain medication (excluding routine postoperative pain pumps), and types of medications used (for previous conditions). The amount of blood loss during the operation and whether painkillers were used after the operation were obtained from the medical records.

#### 2.2.2 Vulnerable elders survey (vulnerable elders survey, VES-13)

Developed by Saliba et al. (9), this tool is used to identify frailty in elderly cancer patients. It has high sensitivity and specificity for cancer patients and has been proven to be an effective and reliable screening tool for frailty in cancer patients. Wu Jun et al. (10) translated this scale into Chinese in 2019, which includes four aspects: age, self-rated health status, physical function, and daily living function, with a total of 13 items. The total score is 10 points, with age scoring 0–3 points, self-rated health scoring 0–1 point, physical function scoring 0–2 points, and daily living function scoring 0–4 points. A score >3 indicates frailty, with higher scores indicating more severe frailty. The Cronbach’s α coefficient is 0.813.

#### 2.2.3 Karnofsky performance status scale (Karnofsky performance status scale, KPS)

This scale was proposed by Karnofsky et al. (11) to assess the physical function and health status of cancer patients. It consists of 10 items, with scores ranging from 0 to 100 (0, 10, 20, 100), with higher scores indicating better health status. A score of 80 or above indicates an independent level, meaning the patient can manage daily activities independently; a score between 50 and 70 indicates a semi-independent level, meaning the patient can manage daily activities with some assistance; a score below 50 indicates a dependent level, meaning the patient requires assistance from others for daily activities. A score above 80 indicates a better postoperative condition and good ability to perform activities of daily living. The Cronbach’s α coefficient is 0.721.

#### 2.2.4 Sleep dysfunction rating scale (sleep dysfunction rating scale, SDRS)

This scale is used to assess patients’ insomnia after surgery. It was developed by Xiao Wei dong (12) and is used to evaluate patients’ sleep patterns over a 3- to 7-day period, covering the overall severity of insomnia and various clinical manifestations. The scale consists of 10 items, using a 5-point rating scale (0–4 points). Higher scores indicate more severe sleep disorders, with a score of ≥14.5 indicating the presence of sleep disorders. The scale has good reliability and validity, with a Cronbach’s α coefficient of 0.890.

#### 2.2.5 Social support rating scale (social support rating scale, SSAS)

The Social Support Rating Scale developed by Xiao Shuiyuan (13) was used, which includes three dimensions: objective support, subjective support, and utilization of social support, comprising a total of 10 items. The score is the sum of the 10 items, assessing the level of social support received by the individual. The total score ranges from 12 to 66 points. A score below 22 indicates low levels of social support, 23–45 indicates moderate levels, and above 45 indicates high levels of social support. Higher scores indicate greater levels of social support. The Cronbach’s alpha coefficient for the scale is 0.825.

#### 2.2.6 Self-rating anxiety scale (self-rating anxiety scale, SAS)

This is an internationally recognized tool for self-assessment of anxiety symptoms, developed by William W. K. Zung (14). It consists of 20 items, with items 5, 9, 13, 17, and 19 scored in reverse, while the remaining items are scored in the forward direction. It uses a 4-point Likert scale, with scores ranging from 1 to 4 for “never or rarely” to 4 to 1 for “most or all of the time.” The total score is calculated by summing the scores of all items, multiplying by 1.25, and rounding to the nearest whole number. A score below 50 is considered normal, 50–59 indicates mild anxiety, 60–69 indicates moderate anxiety, and above 69 indicates severe anxiety. A higher total score indicates greater anxiety in the patient. The Cronbach’s alpha coefficient for the scale is 0.931.

### 2.3 Data collection

This study employed paper-based questionnaires, which were administered by two pre-trained researchers in the relevant departments. Prior to the survey, the research objectives, methods, and significance were explained to the patients. General information was obtained on the day of admission; intraoperative blood loss and pain medication usage were obtained from postoperative medical records; the Frailty Assessment Scale, KPS Functional Status Scale, Sleep Disorder Scale, Social Support Assessment Scale, and Anxiety Self-Rating Scale were assessed on the fourth day postoperatively; the Frailty Assessment Scale was used to assess preoperative frailty within 1–2 days of admission. Completed questionnaires were collected on-site, reviewed for completeness, and ensured to be intact. Data were entered after being verified by a third party.

### 2.4 Date analysis

All data were analyzed using the SPSS 26.0 statistical software to process the collected information. Descriptive statistical analysis was conducted, wherein measurement data were expressed as mean ±standard deviation and count data were represented as frequency or percentage. Comparisons between the two groups were performed using either the chi-square test or the rank sum test, depending on the nature of the data. Univariate analysis was employed to identify potential factors associated with frailty levels, followed by binary logistic regression analysis to determine the predictors of postoperative frailty. A *p*-value less than 0.05 was considered statistically significant.

## 3 Results

### 3.1 Analysis of demographic information, KPS function, sleep dysfunction, social support, and anxiety scores in prostate cancer patients after surgery

Among the 306 patients, the mean age was 72.67 ± 4.97 years, and the mean body mass index (BMI) was 24.62 ± 2.19 kg/m^2^. The average intraoperative blood loss was 62.16 ± 64.12 ml. With regard to marital status, 303 patients (99.02%) were married and 3 (0.98%) were widowed. In terms of educational attainment, 61 patients (19.9%) had completed primary school, 97 (31.7%) junior high school, 91 (29.7%) senior high school, 8 (2.6%) technical secondary school, 32 (10.5%) college, and 17 (5.6%) held a bachelor’s degree. A total of 304 patients (99.35%) had medical insurance coverage, while 2 (0.65%) did not. Regarding physical activity, 17 patients (5.6%) reported no regular exercise, 71 (23.2%) exercised 1–2 times per week, 65 (21.1%) exercised 3–4 times per week, and 153 (50.0%) exercised 6–7 times per week. In terms of comorbidities, 278 patients (90.0%) had 0–2 pre-existing chronic conditions, while 28 (9.1%) had three or more. Medication use included 60 patients (19.6%) who took no medications, 178 (58.2%) who used 1–3 types, and 68 (22.2%) who used 4–6 types. A history of falls within the past year was reported by 27 patients (8.8%), whereas 279 (91.2%) reported no such history. With respect to lifestyle factors, 73 patients (23.9%) were current smokers and 233 (76.1%) were non-smokers; 56 (18.3%) consumed alcohol regularly, while 250 (81.7%) did not. Fifty-three patients (17.3%) had undergone surgery within the previous year, compared to 253 (82.7%) who had not. Postoperative analgesic use (excluding routine patient-controlled analgesia pumps) was documented in 4 patients (1.3%), with 302 (98.7%) not receiving additional pain medication. The mean Karnofsky Performance Status score was 63.73 ± 9.12; the mean Sleep Disorder Scale score was 10.86 ± 4.46; the Social Support Rating Scale yielded a mean score of 38.18 ± 5.41; the Anxiety Self-Rating Scale score averaged 36.40 ± 4.77 (Table 1).

**Table 1:** General information, KPS function, sleep disorders, social support, and anxiety scores.

Variables	Category	*n* (%)	$\bar{x}$ *±s*
Age (years, $\bar{x}$ *±s*)			72.67 ± 4.97
BMI (Kg/m^2^, $\bar{x}$ *±s*)			24.62 ± 2.19
Intraoperative Blood Loss			62.16 ± 64.12
Marital status, *n* (%)	Married	303 (99.0)	-
	Widowed	3 (1.0)	-
Education level, *n* (%)	Elementary school	61 (19.9)	-
	Junior High School	97 (31.7)	-
	High School	91 (29.7)	-
	Vocational School	8 (2.6)	-
	Associate Degree	32 (10.5)	-
	Bachelor’s Degree	17 (5.6)	-
Medical Payment Method, *n* (%)	No insurance	2 (0.7)	-
	With insurance	304 (99.3)	-
Frequency of daily exercise, *n* (%)	Never	17 (5.6)	-
	1–2 times/week	71 (23.2)	-
	3–4 times/week	65 (21.2)	-
	6–7 times/week	153 (50.0)	-
Number of previous diseases, *n* (%)	0–2	278 (90.9)	-
	> = 3	28 (9.1)	-
Types of medication, *n* (%)	No medication	60 (19.6)	-
	1–3 types	178 (58.2)	-
	4–6 types	68 (22.2)	-
History of falls, *n* (%)	Yes	27 (8.8)	-
	No	279 (91.2)	-
History of smoking, *n* (%)	Yes	73 (23.9)	-
	No	233 (76.1)	-
Drinking history, *n* (%)	Yes	56 (18.3)	-
	No	250 (81.7)	-
Surgical history within 1 year, *n* (%)	Yes	53 (17.3)	-
	No	253 (82.7)	-
Postoperative use of analgesics, *n* (%)	Yes	4 (1.3)	-
	No	302 (98.7)	-
Karnofsky Performance Status (Score)			63.73 ± 9.12
Sleep Disturbance Score (Score)			10.86 ± 4.46
Social Support Level (Score)			38.18 ± 5.41
Anxiety Score (Points)			36.40 ± 4.77

$\bar{x}$ ±s: mean±Standard deviation.

### 3.2 Postoperative frailty in prostate cancer patients

**Figure 1:**
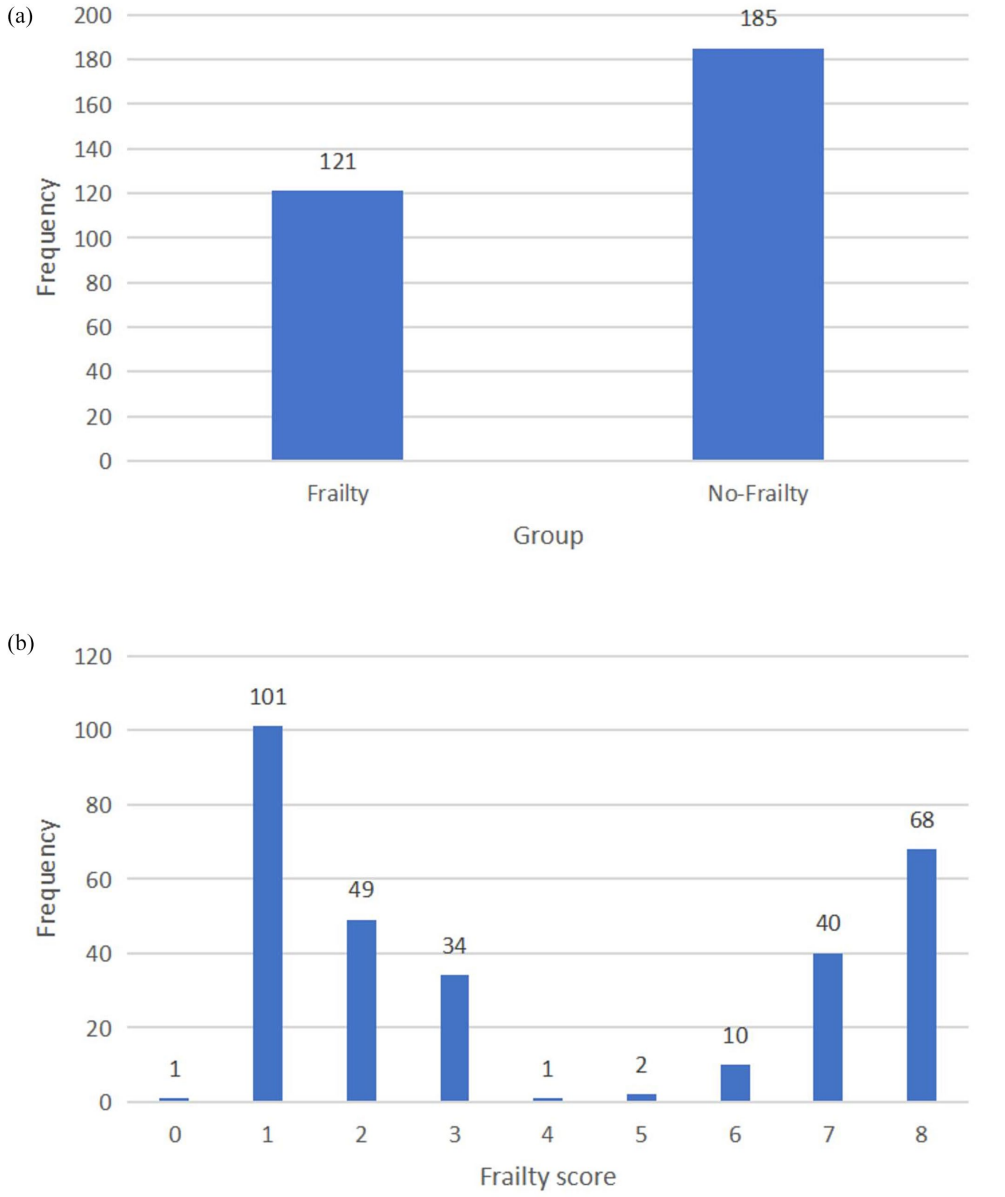
**(a)** Histogram of frailty scores for different groups. **(b)** Histogram of the scores for weakness.

According to the frailty assessment criteria of the VES-13 scale, a total score > 3 is defined as frailty. Among 306 postoperative prostate cancer patients, the incidence of postoperative frailty was 39.5% (121/306), and the incidence of non-frailty was 60.5% (185/306; Table 2; Figures 1a,b).

**Table 2:** Incidence of postoperative frailty in prostate cancer patients.

Variables	Category	Number of people	Proportion (%)
VES-13 Score	<=3	185	60.5
	>3	121	39.5

### 3.3 Univariate analysis of postoperative frailty in prostate cancer patients

The results showed that there were no statistically significant differences between the frailty group and the non-frailty group in terms of marital status, method of medical payment, types of medication, history of falls within 1 year, smoking, drinking, history of surgery within 1 year, and use of pain medication (*p* > 0.05). Statistically significant differences were observed in age, BMI, intraoperative blood loss, educational level, number of pre-existing conditions, frequency of daily exercise, KPS functional status, sleep disorders, social support, and anxiety (*p* < 0.05) (Table 3).

**Table 3:** Univariate analysis of postoperative frailty in prostate cancer patients.

Variables	Category	Non-frail (*n* = 185) [*n* (%)]	Frail (*n* = 121) [*n* (%)]	*Z/X* ^2^	*p*
Age [years, M(P25, P75)]		72 (72, 73)	75 (73, 78)	−8.616^1)^	<0.001
BMI [Kg/m^2^, M(P25, P75)]		24.09 (23.16,25.35)	24.61 (23.15,25.95)	−2.862^1)^	0.004
Intraoperative blood loss [ml, M(P25, P75)]		50 (30,50)	60 (60,100)	−12.450^1)^	<0.001
Marital status, *n* (%)				0.932	0.334
	Married	1 (0.5)	2 (1.7)		
	Widowed	184 (99.5)	119 (98.3)		
Education level, *n* (%)				21.926	0.001
	Elementary school	34 (18.4)	27 (22.3)		
	Junior High School	48 (25.9)	49 (40.5)		
	High School	70 (37.8)	21 (17.4)		
	Vocational School	3 (1.6)	5 (4.1)		
	Associate Degree	23 (12.4)	9 (7.4)		
	Bachelor’s Degree	7 (3.8)	10 (8.3)		
Medical Payment Method, *n* (%)				0.092	0.762
	No insurance	1 (0.5)	1 (0.8)		
	With insurance	184 (99.5)	120 (99.2)		
Exercise frequency, *n* (%)				114.997	<0.001
	Never	6 (3.2)	11 (9.1)		
	1–2 times/week	8 (4.3)	63 (52.1)		
	3–4 times/week	41 (22.2)	24 (19.8)		
	6–7 times/week	130 (70.3)	23 (19.0)		
Number of previous diseases, *n* (%)				20.805	<0.001
	0–2	150 (81.1)	69 (57.0)		
	> = 3	35 (18.9)	52 (43.0)		
Types of medication, *n* (%)				5.115	0.078
	No medication	33 (17.8)	27 (22.3)		
	1–3 types	117 (63.3)	61 (50.4)		
	4–6 types	35 (18.9)	33 (27.3)		
History of falls in the past year, *n* (%)				0.018	0.894
	Yes	16 (8.6)	11 (9.1)		
	No	169 (91.4)	110 (90.9)		
History of smoking, *n* (%)				0.056	0.812
	Yes	45 (24.3)	28 (23.1)		
	No	140 (75.7)	93 (76.9)		
Drinking history, *n* (%)				0.002	0.965
	Yes	34 (18.4)	22 (18.2)		
	No	151 (81.6)	99 (81.8)		
Surgical history within 1 year, *n* (%)				0.088	0.767
	Yes	33 (17.8)	20 (16.5)		
	No	152 (82.2)	101 (83.5)		
Postoperative use of analgesics, *n* (%)				2.131	0.144
	Yes	1 (0.5)	3 (2.5)		
	No	184 (99.5)	118 (97.5)		
Karnofsky Performance Status [score, M(P25, P75)]		70 (60,70)	60 (50,60)	−11.182^1)^	<0.001
Sleep Disturbance Score [score, M(P25, P75)]		9 (7,11)	14 (11,17.5)	−9.160^1)^	<0.001
Social support level [score, M(P25, P75)]		41 (39,42)	34 (31,36)	−11.906^1)^	<0.001
Anxiety score [score, M(P25, P75)]		33 (32,35)	39 (38,41)	−12.724^1)^	<0.001

1. Wilcoxon rank-sum test 2. M(P25, P75): Median.

### 3.4 Logistic regression analysis of factors influencing postoperative frailty in prostate Cancer patients

After screening each variable through univariate analysis, variables with statistical differences (*p* < 0.05) were assigned values (Table 4). Binary logistic regression analysis revealed that age, intraoperative blood loss, Karnofsky functional status score, social support level, anxiety status and exercise frequency (≥3 times per week) are significant influencing factors for postoperative frailty in prostate cancer patients (*p* < 0.05). Increasing age is associated with a higher risk of postoperative frailty in prostate cancer patients (OR = 1.453, 95% *CI*: 1.108 ~ 1.905, *p* = 0.007); The risk of postoperative frailty in prostate cancer patients exhibits a positive correlation with intraoperative blood loss volume. (OR = 1.192, 95% *CI*: 1.072 ~ 1.326, *p* = 0.005); higher Karnofsky functional status scores after surgery are associated with a lower risk of frailty in prostate cancer patients (OR = 0.739, 95% *CI*: 0.602 ~ 0.906, *p* = 0.004); higher social support levels after surgery are associated with a lower risk of frailty in prostate cancer patients (OR = 0.478, 95% *CI*: 0.307 ~ 0.743,*p* = 0.001); increased anxiety levels are associated with an increased risk of frailty (OR = 1.623, 95% *CI*: 1.173 ~ 2.246, *p* = 0.003); Compared to non-exercising individuals, prostate cancer patients who engaged in regular exercise (≥3 times per week) demonstrated a significantly reduced risk of postoperative frailty.(OR = 0.071, 95% *CI*: 0.006 ~ 0.861, *p* = 0.038; OR = 0.079, 95%*CI*: 0.007 ~ 0.927, *p* = 0.043) (Tables 5).

**Table 4:** Logistic regression analysis independent variable assignment table.

Variable	Assignment Method
Age	Original value input
BMI	Original value input
Intraoperative blood loss	Original value input
Education level	Primary school = 0, Junior high school = 1, High school = 2, Technical secondary school = 3, College = 4, Bachelor’s degree = 5
Number of previous diseases	0 ~ 2 types = 0, > = 3 types = 1
Frequency of daily exercise	Never = 0, 1 ~ 2 times/week = 1 3 ~ 4 times/week = 2, 6 ~ 7 times/week = 3
Karnofsky functional status score	Original value input
Sleep disorder score	Original value input
Social support level score	Original value input
Anxiety status score	Original value input

**Table 5:** Binary logistic regression analysis of influencing factors for postoperative frailty in prostate cancer patients.

Independent variable	Regression coefficient	SD	*Wald X^2^*	*P*	OR	95% *CI*
Age	0.374	0.138	7.304	0.007	1.453	1.108 ~ 1.905
BMI	−0.158	0.183	0.740	0.390	0.854	0.596 ~ 1.224
Intraoperative blood loss	0.176	0.054	10.503	0.001	1.192	1.072 ~ 1.326
Education level(primary school as a reference)	-	-	5.711	0.335	-	-
Education Level (Junior High School)	0.057	2.261	0.001	0.980	1.059	0.013 ~ 88.990
Education Level (High School)	−1.481	2.188	0.458	0.499	0.228	0.003 ~ 16.577
Education Level (Vocational School)	1.039	2.443	0.181	0.671	2.826	0.024 ~ 339.630
Education Level (Associate Degree)	−1.103	2.702	0.167	0.683	0.332	0.002 ~ 66.238
Education level (Bachelor’s)	−2.347	2.426	0.935	0.333	0.096	0.001 ~ 11.122
Number of previous diseases	−0.442	1.111	0.158	0.691	0.643	0.073 ~ 5.670
Exercise Frequency(Never as reference)	-	-	6.513	0.089	-	-
Exercise Frequency (1 ~ 2 times/week)	−3.118	1.61	3.752	0.053	0.044	0.002 ~ 1.037
Exercise frequency (3 ~ 4 times/week)	−2.650	1.276	4.317	0.038	0.071	0.006 ~ 0.861
Exercise frequency (6 ~ 7 times/week)	−2.540	1.257	4.080	0.043	0.079	0.007 ~ 0.927
Karnofsky functional status score	−0.303	0.104	8.441	0.004	0.739	0.602 ~ 0.906
Sleep disorder score	0.211	0.133	2.541	0.111	1.235	0.953 ~ 1.602
Social support level score	−0.738	0.225	10.750	0.001	0.478	0.307 ~ 0.743
Anxiety status score	0.484	0.166	8.555	0.003	1.623	1.173 ~ 2.246

## 4 Discussion

### 4.1 The incidence of postoperative frailty in prostate cancer patients is relatively high

In this study, among 306 patients who underwent radical prostatectomy for prostate cancer, the incidence of postoperative frailty was 39.5%, which was similar to the results reported by Meissner et al. (15) and slightly lower than the results reported by Tohi et al. (6). This study used the VES-13 Frailty Scale to assess patients with stage I-II disease who underwent surgery alone, while Tohi et al. used the Frailty Index for assessment, and the study sample included all patients who underwent surgery and chemoradiotherapy. It can be seen that different assessment tools and assessment populations can lead to certain differences in results (16). Currently, the assessment of frailty in patients after prostate cancer surgery mostly uses general scales supplemented by cancer-related indicators. There are no standardized and targeted frailty screening tools for assessing frailty after prostate cancer surgery. Therefore, in the future, it may be possible to develop a comprehensive frailty assessment form for patients after prostate cancer surgery, with the aim of providing medical staff with more accurate screening and assessment tools.

The choice of different postoperative assessment time points may also lead to variations in the incidence of frailty. Yamada et al. (17) assessed 1,063 patients who underwent surgery on postoperative day 1, finding a frailty incidence of 62%, which was higher than the result measured on postoperative day 4 in this study. The reason may be that assessments on the first postoperative day are influenced by acute surgical inflammation, metabolic disturbances, and various medications, leading to stress responses being misclassified as debilitated states and resulting in an overestimation of the true prevalence of debilitation. Liu et al. (18) conducted frailty assessments on postoperative day 4, reporting a frailty prevalence of 38%, which is comparable to the findings of this study. Furthermore, after constructing a predictive model, it was discovered that this time point showed the highest correlation with the prediction of activities of daily living at 3 months postoperatively. Zhao et al. (19) suggest that assessing frailty on postoperative day 4 yields a lower incidence compared to earlier time points. By this stage, acute inflammation begins to subside, functional indicators gradually recover, and patients are sufficiently alert to accurately complete subjective questionnaire items. This timing better reflects reversible frailty associated with surgical stress. Therefore, the fourth postoperative day can serve as a critical assessment point, aligning with the clinical intervention window while avoiding premature over diagnosis. Some studies (20) suggest that frailty is a dynamic developmental process that may only gradually manifest in the long-term postoperative period, necessitating continuous follow-up to avoid the risk of misdiagnosis. This study was evaluated at a single time point only; future research should be further refined through multi-time-point designs.

Compared with the incidence of postoperative weakness in patients with other common cancers (21, 22), The results of this study found that the incidence of postoperative frailty in prostate cancer patients was slightly higher than that in other cancer patient groups. The analysis of the reasons revealed that, compared to the wide age distribution of other cancers, prostate cancer patients tend to be older, with most being over 70 years old. The significant decline in physical function in elderly patients can accelerate the onset of postoperative frailty (23). Therefore, during postoperative care, it is important to actively monitor patients for signs of frailty and to strengthen continuous dynamic assessment of their physical condition in order to identify signs of frailty as early as possible, provide targeted interventions, and effectively prevent and improve frailty.

### 4.2 Factors influencing postoperative frailty in prostate cancer

#### 4.2.1 The older the patient, the more severe the postoperative weakness in patients

The results of this study show that older prostate cancer patients have higher levels of postoperative frailty. In recent years, many foreign scholars have conducted research on the factors affecting postoperative frailty in prostate cancer patients, all of which indicate that age is an important factor in inducing postoperative frailty in tumor patients, and the progression of frailty further exacerbates the occurrence and development of the disease (4), for every additional year of age, the risk of postoperative weakness increases by 1.65 times (24). Boozari et al. also confirmed that there is a linear relationship between age and frailty, and that increasing age in elderly cancer patients is positively correlated with an increased risk of frailty (25), which is consistent with the results of this study. As we age, mitochondrial function in cells gradually declines, and the cell apoptosis cycle shortens. These changes lead to functional decline and accelerate the aging process. Additionally, cancer patients often have chronic low-grade inflammation, and surgical trauma further exacerbates inflammatory responses, leading to reduced antioxidant capacity and elevated pro-inflammatory cytokines (26). This not only exacerbates bodily but also interacts with the age-related physiological decline mechanisms mentioned above, jointly inducing or exacerbating postoperative frailty. Although age is an uncontrollable objective factor, given its close association with frailty, an increasing number of researchers are exploring its pathophysiological mechanisms to better predict frailty risk from an age-related perspective.

#### 4.2.2 The greater the amount of intraoperative bleeding, the more severe the postoperative weakness

The results of this study show that patients with greater intraoperative blood loss experience greater postoperative frailty. From a physiological perspective, the direct impact of intraoperative blood loss on the body is one of the core factors contributing to frailty (4). Massive blood loss can cause a sudden decrease in effective circulating blood volume, which directly affects tissue perfusion and cellular hypoxia. This can lead to a systemic stress response, accelerate skeletal muscle catabolism, and cause nutritional absorption disorders after surgery, resulting in significant muscle mass loss and physical decline. At the same time, blood loss can easily cause anemia in elderly patients, reducing the oxygen-carrying capacity of hemoglobin. A multicenter study found that hemoglobin concentration is an important predictor of postoperative frailty (27), consistent with the results of this study. Postoperative patients with anemia typically exhibit symptoms of frailty, including fatigue and reduced exercise tolerance. Studies have shown that for every 100 ml increase in intraoperative blood loss in elderly patients, the risk of delayed recovery of physiological reserves within 1 week postoperatively increases by 15% (28). This can impair tolerance for early postoperative physical exercise rehabilitation, creating a closed-loop cycle of “blood loss-anemia-reduced activity-muscle atrophy.” Therefore, preoperative assessment of vascular fragility and coagulation function in surgical patients is recommended, along with the use of precise hemostasis techniques to minimize blood loss postoperatively. Early postoperative dynamic monitoring of changes in hemoglobin concentration levels and trends should be conducted, and nutritional support should be strengthened to prevent the onset of anemia.

#### 4.2.3 The higher the Karnofsky performance status score, the lower the risk of frailty

The Karnofsky Performance Status Score directly reflects the physiological reserve capacity of the body. The results of this study showed that the incidence of frailty in patients with low performance status scores was 0.739 times that of patients with high scores, which is similar to the results of Cadwell et al. (29). The analysis of the reasons shows that, first, patients with high functional status have higher rehabilitation initiative and compliance. For patients who have undergone prostate cancer surgery, postoperative urinary incontinence is the biggest obstacle to activity. However, patients with good functional status are more likely to understand the importance of postoperative rehabilitation and are more tolerant of the discomfort caused by rehabilitation, thereby adhering to the rehabilitation exercise plan and preventing frailty. Secondly, the decline in postoperative functional status often progresses concurrently with the occurrence of various complications (30). Patients with better functional status can leverage their stronger bodily repair and compensatory capabilities to activate the body’s self-recovery mechanisms (31), thereby reducing the occurrence of pre-frailty symptoms such as decreased postoperative mobility and lowering the probability of frailty progression. Additionally, patients with higher functional status have a stronger sense of control over their own bodies. When faced with surgical procedures and postoperative discomfort, they can exhibit stronger self-regulation in response to anxiety, depression, and other negative emotions. Healthy psychological well-being and a positive attitude toward illness are crucial factors in delaying frailty. Therefore, healthcare providers can use the KPS functional status score as an important predictive indicator for postoperative frailty. For patients with lower scores, intervention plans such as nutritional or exercise interventions can be developed in the early postoperative period to reduce the risk of frailty. In the future, the KPS functional status score can be further refined to include more comprehensive assessment indicators, providing a basis for precise interventions.

#### 4.2.4 A higher exercise frequency helps reduce the risk of postoperative frailty

The results of this study showed that patients who exercised intensively every week had a significantly lower risk of frailty (*p* < 0.05), which was consistent with the results of studies by Angulo et al. (32) and Li Huanhuan et al. (33). Research indicates that regular exercise is a key strategy for improving postoperative frailty, suggesting that planning the frequency of exercise for postoperative patients can effectively mitigate the risk of frailty. Park et al. (34) conducted a study on patients who underwent radical prostatectomy, implementing a multi-component exercise program based on pelvic floor muscle exercises combined with aerobic and resistance training. After a 12-week follow-up, the primary outcome measure was frailty, with secondary outcome measures including exercise capacity, urinary incontinence severity, and quality of life. The study analyzed the association between exercise and these three outcome measures. The results showed that the frailty scores in the intervention group were significantly lower than those in the control group after 12 weeks of regular exercise. Additionally, the intervention group demonstrated superior exercise capacity, urinary incontinence severity, and quality of life compared to the control group. A meta-analysis (35) also confirmed that prostate cancer benefits more from high-frequency exercise than other cancers. Based on these findings, exercise frequency can be incorporated into perioperative management in clinical practice. Exercise manuals can be provided to patients in the early postoperative period to explain relevant exercise knowledge. Remote online education can be conducted through online media platforms to promote healthy lifestyles and exercise habits, ensuring the safety and continuity of exercise to indirectly reduce the incidence of frailty.

#### 4.2.5 Low social support levels and high anxiety levels are risk factors for frailty

The results of this study indicate that high levels of social support are a protective factor against frailty in patients who have undergone prostate cancer surgery, while high levels of anxiety are a risk factor for frailty in these patients, which is consistent with the results of previous studies (36, 37). High levels of social support help patients obtain emotional, material, and informational support from their families, friends, healthcare providers, and social networks through various channels. These forms of support collectively form a protective network that can alleviate patients’ concerns about their prognosis after surgery, compensate for their need for emotional comfort and lack of professional knowledge (26), and help them better cope with feelings of helplessness and loneliness after surgery, thereby preventing negative emotions from triggering frailty. Research has confirmed that low levels of social support and high anxiety are independent factors contributing to frailty, and that the two also interact with each other (38). On the one hand, when individuals face stress and difficulties, a lack of effective social support makes it difficult for them to express their negative emotions, leading to a continuous accumulation of negative emotions. On the other hand, high anxiety levels cause postoperative patients to have a more negative perception of the future, making it difficult for them to effectively utilize the support available to them and accelerating the onset of frailty. On the other hand, postoperative patients with low levels of social support may find it difficult to maintain healthy behaviors, and high anxiety can further inhibit the implementation of healthy behaviors, leading to a decline in physical function and increasing the risk of frailty. Therefore, assessments of social support and anxiety levels can be used as targets for frailty prevention and intervention, while leveraging their interrelatedness to develop coordinated intervention measures for postoperative frail patients.

## 5 Conclusion

This study clearly indicates that one-third of prostate cancer patients experience weakness symptoms after surgery, which has become a common postoperative problem that requires high attention. The identified core influencing factors include age, intraoperative blood loss, KPS functional status, social support, and anxiety state. The core clinical significance lies in providing a practical and multi-dimensional management direction for clinical practice: by implementing dynamic monitoring for different risk groups (such as the elderly and those with excessive intraoperative blood loss), providing nutritional and exercise interventions for patients with poor function, offering psychological counseling to anxious patients, and strengthening social support, it is possible to effectively reduce the risk of postoperative weakness, thereby effectively assisting in optimizing the postoperative care plan and improving patient prognosis. Although the scope of factors covered in this study may not be comprehensive, and no random sampling or multi-center data collection was adopted, it still provides important clinical evidence for the management of postoperative weakness in prostate cancer. In the future, by expanding the variable range and adopting a multi-center design and random sampling method, the comprehensiveness and reliability of the study will be further enhanced.

## Data Availability

The original contributions presented in the study are included in the article/supplementary material, further inquiries can be directed to the corresponding author.
